# Influence of Curing Temperature on the Synthesis of a Phosphate-Waste-Based Geopolymer for CO_2_ Capture and Separation

**DOI:** 10.1021/acsaem.5c00426

**Published:** 2025-06-03

**Authors:** Mariana Schneider, Denise Gomes da Silva Costa, Enrique Rodríguez-Castellón, M. Olga Guerrero-Pérez, Dachamir Hotza, Agenor De Noni, Regina de F. P. M. Moreira

**Affiliations:** † Department of Chemical Engineering and Food Engineering, 28117 Federal University of Santa Catarina, 88040-900 Florianópolis, Santa Catarina, Brazil; ‡ Department of Inorganic Chemistry Faculty of Sciences, University of Málaga, E29071 Málaga, Spain; § Department of Chemical Engineering, Faculty of Sciences, University of Málaga, E29071 Málaga, Spain

**Keywords:** geopolymer, curing temperature, submerged cure, phosphate waste, CO_2_ adsorption

## Abstract

A promising approach to combating global warming is the capture, storage, and reuse of greenhouse gas emissions. Adsorption processes can capture up to 90% of the CO_2_ emissions. However, CO_2_ storage requires significant investment and may not always be feasible, making it essential to improve capture efficiency and reduce costs. Geopolymeric adsorbents have shown potential for separating CO_2_ from gaseous mixtures. Additionally, captured CO_2_ can be utilized in processes such as electrochemical reduction, photocatalytic reduction, and catalytic methanation. This study compares the potential of four geopolymers synthesized from phosphate waste and metakaolin as precursors under different curing conditions by analyzing the adsorption equilibrium isotherms of CO_2_, H_2_, and CO at various temperatures. The samples were characterized using XRF, XRD, FTIR, SEM, EDS, XPS, NMR, micro-CT, density, BET surface area, and porosity analyses. The best performance was observed for the submerged samples, which exhibited a CO_2_ adsorption capacity of 2.24 mmol/g at 80 °C and 2.00 mmol/g at 65 °C, highlighting the significance of the submerged-cured process. BET surface area analysis revealed values of 301 and 337 m^2^/g for the submerged G65s and G80s samples, with corresponding porosity values of ∼0.130 cm^3^/g. Additionally, FTIR and NMR analyses confirmed the successful geopolymerization and identified key aluminosilicate peaks. These findings highlight the potential of industrial waste-based geopolymers as sustainable and cost-effective adsorbents for CO_2_ separation, with key characteristics such as reusability and compatibility with cyclical processes further enhancing their suitability for practical applications in gas separation technologies.

## Graphical abstract

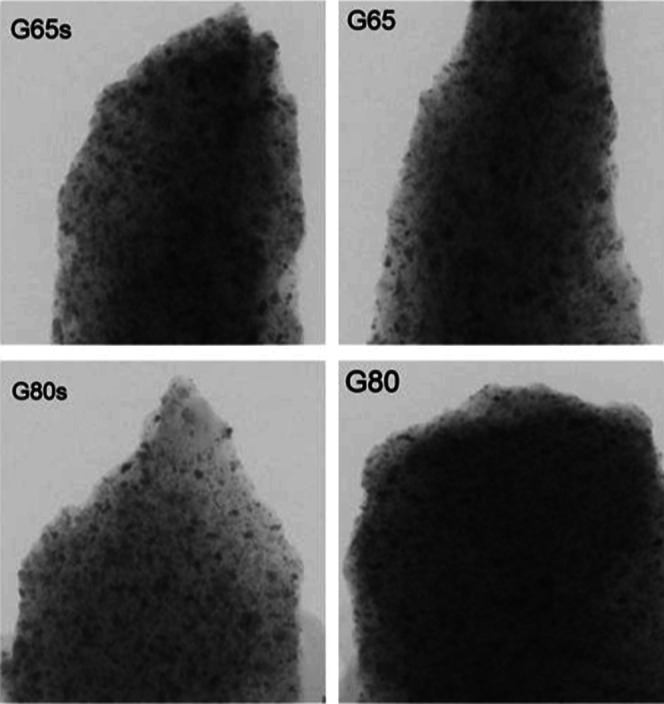

## 1 Introduction

Fossil fuels have been the world’s main source of energy and are expected to remain so for several decades. Their combustion is responsible for greenhouse gas emissions that cause permanent and irreversible damage to the climate system.^1,2^ There are two main options for reducing the presence of these emissions: replacing fossil energy sources with renewable fuels and capturing carbon dioxide (CO_2_) from the atmosphere. Considering the obstacles to achieving the first option, the capture, storage, and use of greenhouse gases play a crucial role in reducing the carbon footprint of energy production and combating global warming.^3^

It is estimated that around 90% of CO_2_ emissions can be captured and stored after the separation process.^4^ Exhaust gases from industrial processes and power plants are among the primary sources of CO_2_ emissions; therefore, their treatment is essential to mitigate the release of this greenhouse gas. These emissions typically come from flue gases in power generation, cement production, and various industrial processes, where CO_2_ concentrations range between 5% and 15% at near-ambient pressure. However, high capital cost and limited storage options require the use of the captured carbon dioxide.^4^ Thus, it is necessary to improve the capture of CO_2_ and its conversion into usable compounds such as methane, methanol, and dimethyl ether, among others.^1,3^

Despite extensive research on CO_2_ capture, there is a gap in the development of low-cost, reusable, and efficient adsorbent materials for CO_2_ separation capable of being integrated into cyclical processes, further enhancing their suitability for practical applications in gas separation technologies. In many processes, CO_2_ must be separated from gases like N_2_, H_2_, or CH_4_.^5^ Also recently, there has been a considerable increase in research on adsorbent materials used for CO_2_ adsorption. In this regard, aluminosilicates like zeolites have been extensively studied in CO_2_ separation due to their crystalline and microporous characteristics, high surface area, thermal stability, and adsorption selectivity.^5^ However, zeolites often have limitations such as their narrow pore size, which can hinder their performance in separating CO_2_ from mixtures containing molecules like CH_4_ or N_2_.^6^ In contrast, geopolymers, or geopolymer/zeolite hybrids, offer a more versatile solution due to their adaptable structure, high surface area, mechanical and chemical resistance, and low synthesis costs, making them particularly suitable for gas separation processes.^7^

Compared to other adsorbents, such as MOFs (metal–organic frameworks)^8^ and activated carbons,^9^ geopolymers stand out due for their cost-effectiveness and durability. While MOFs offer high CO_2_ selectivity, they tend to be more expensive, and their stability under humidity, heat, and mechanical stress requires thorough assessment for industrial use.^10^ Activated carbons, although commonly used, show limited selectivity under humid conditions. A study involving bamboo-derived activated carbon demonstrated a 75% decrease in CO_2_ adsorption capacity when exposed to water vapor.^11^ Geopolymers, with their unique combination of stability and customizable properties, offer a competitive and balanced alternative to CO_2_ capture. This advantage stems from their distinctive material structure and synthesis process.

Geopolymers are amorphous three-dimensional aluminosilicate materials, classified as inorganic polymers, with ceramic-like properties and are produced and hardened at ambient temperature. The geopolymerization process is an exothermic phenomenon and occurs under highly alkaline conditions. Reactive aluminosilicates dissolve quickly and free SiO_4_^–^ and AlO_4_^–^ tetrahedra units are released into the solution.^6,12^ The result of the polycondensation of monomers is referred to as orthosilicates, an abbreviation for silicon-oxo-aluminate.^13^

Porous geopolymers have gained much attention due to the flexibility of their production processes and the diversity of their structural properties.^14^ It is possible to control their porosity and morphology without losing the essential characteristics of the mechanical resistance of these materials, allowing their application to be expanded to a wide variety of fields. The application of geopolymers as adsorbents can be improved by developing a hierarchical porous in the material.^14,15^

The materials used in geopolymer synthesis are mainly aluminosilicate sources. As eco-friendly options, several waste materials have been investigated such as civil construction waste, ground brick waste powder,^16^ coconut ash,^17^ fly ash,^18,19^ rice husk ash, mineral coal,^18^ slag, pozzolan, clay,^13^ phosphate waste,^20–22^ and metakaolin/kaolinite.^14,18,22–25^ Due to their easy preparation and the diversity of the raw materials, it is not surprising that geopolymerization is considered a suitable way to add value to solid industrial residues, which are low-cost and whose use helps reduce environmental waste impacts.

Therefore, this study aims to address this gap by synthesizing and characterizing a geopolymer using metakaolin and phosphate waste as precursors and evaluating its CO_2_ adsorption potential to capture CO_2_ from exhaust gases through equilibrium adsorption isotherms carried out at various temperatures and pressures. These materials exhibit favorable characteristics for a range of energy-focused applications. Notably, they could be employed in the advancement of blue hydrogen technology, which involves producing hydrogen from natural gas alongside carbon dioxide capture.^72^

## 2 Materials and Methods

### 2.1 Materials

Kaolin was obtained from CAULISA—Indústria de Caulim S.A (Pernambuco, Brazil), phosphate waste (PW) mining tailings were provided by a mine in Goiás (Brazil), sodium hydroxide (NaOH, micropearls with 98.5% purity) was purchased from VWR chemicals, and the sodium silicate solution (SS)—SiO_2_/Na_2_O (molar ratio SiO_2_/Na_2_O: 2.5) was obtained from Sigma-Aldrich.

### 2.2 Geopolymer Synthesis

The geopolymer was synthesized based on Freire et al. (2024), Minelli et al. (2016), and Chen et al. (2020) using metakaolin and phosphate waste as precursors and a mix of sodium hydroxide (NaOH) and sodium silicate (Na_2_SiO_3_) as an alkaline activator.^18,100,101^ A complete characterization of metakaolin and phosphate waste was carried out by Freire et al. (2024) and Freire et al. (2020). Metakaolin is composed predominantly of silicon, at 54.3%, and iron oxides, at 44.2%,^18^ and the main chemical composition of PW is silicon at approximately 49% and iron oxides at 21%.^22^

To synthesize the metakaolin, the kaolin was first calcined at 900 °C for 60 min. Davidovits et al. (1994) recommend optimal proportions of oxides (SiO_2_, Al_2_O_3_, Na_2_O) and water to achieve a balanced geopolymerization process. These proportions were carefully selected to ensure the development of geopolymers with desirable properties, such as slurry workability, compressive strength, surface area, pore size, pore volume, and minimal efflorescence. Following these recommendations, the molar ratios for the geopolymer synthesis were set as SiO_2_/Al_2_O_3_ = 3.30; Na_2_O/SiO_2_ = 0.30; Na_2_O/Al_2_O_3_ = 0.99; and H_2_O/Na_2_O = 12.00.^27^

The geopolymer was then prepared by following six steps: (i) mixing the two solid components (metakaolin and PW); (ii) preparing the alkaline activator, mixing NaOH, SS, and deionized water (DIW) at 800 rpm for 10 min on a magnetic stirrer; (iii) preparing the suspension by adding the solids to the alkaline activator solution and mixing for 15 min at 1500 rpm, in a mechanical stirrer; (iv) molding the geopolymeric slurry into a cylindrical shape, using a 2 × 4 cm silicone mold; (v) preparing two samples and curing them in an oven (VENTI-line VWR chemicals) at 80 and 65 °C (the mold was covered with a high-temperature tape so water would evaporate slowly); and (vi) submersing part of the samples in DIW after demolding to remove unreacted sodium ions and leaving the other part to cure without submersion, with both samples left at room temperature (25 °C ± 2 °C) for 30 days. Finally, the submersed material was dried in an oven at 100 °C for 24 h. The composition of the samples is presented in Table 1. The geopolymer samples cured at 65 °C were named G65s and G65 for submersed cure and dry cure, respectively. Similarly, the samples cured at 80 °C were named G80s for the submersed cure and G80 for the dry cure.

**Table 1:** Geopolymer Formulation

precursor material	(wt %)
metakaolin	35
phosphate waste	19
NaOH	11
sodium silicate	13
water	22

### 2.3 Characterization

All of the characterizations were made at the Central Research Support Service (SCAI), at the University of Málaga.

The geopolymer samples were analyzed by X-ray fluorescence (XRF) to determine their chemical composition and quantify the oxides using Thermo Fisher Scientific equipment. The density of the samples was evaluated using a pycnometer AccuPyc II1340 with helium, in a 1.0 cm^3^ chamber at 23.33 °C with an equilibration rate of 0.0050 psig/min.

The crystalline structure and mineralogical composition of the samples were evaluated by powder X-ray diffraction (XRPD). The crystalline phases in the materials were identified with X’pert HighScore plus software. The chemical stability of the samples to CO_2_ attack was evaluated by performing XRD analyses before and after exposure to CO_2_. The analysis was carried out using a PANanalytical Empyrean automated diffractometer. Powder patterns were recorded in the Bragg–Brentano reflection configuration using the PIXcel 3D detector with a step size of 0.017° (2θ). The powder patterns were recorded between 4 and 70° in 2θ with a total measuring time of 30 min.

Scanning electron microscopy (SEM) was employed to study the morphology of the materials and conduct chemical analysis. This technique identifies material components and provides detailed insights into the internal structure of the samples including crystal structure, morphology, and stress state information. The equipment used was a FESEM Tescan Clara microscope with a Leica EM ACE600 metallizer. The samples were coated with 4 nm of platinum to make them more conductive for a better analysis.

The BET surface area (m^2^/g) is an important parameter to characterize solids used for processes involving their surface since the kinetics of heterogeneous solid–fluid interaction processes directly depend on the specific area of this solid. The specific surface area, pore size, and total pore volume were obtained through nitrogen adsorption–desorption isotherms at −196 °C using Micromeritics ASAP 2020 equipment.

For microcomputed tomography (μCT), the samples were scanned employing a SkyScan 2214 (Bruker) system. Projections were obtained using a W source filament and a 0.25 or 0.5 mm Al foil (for sample 3 or the other samples, respectively) in front of the CDD3 detector to minimize the beam hardening artifact. This source was set to 45 or 95 kV and 130 or 86 μA (for sample 3 and the other samples respectively). The CCD3 detector with a physical pixel size of 17.427 μm was set at a middle position with a source-to-detector distance of 315.449 mm and a source-to-sample distance of 10.861 or 39.823 mm, which yielded a voxel size of 0.6 μm for sample 3 or 2.2 μm for the other samples. All scans were acquired over 360° (0.15 or 0.2° rotation step) using an exposure time of 2.9 or 1.75 s for sample 3 and the other samples, respectively. This resulted in an overall recording time of 8.5 or 4.5 h per data set (for sample 3 and the other samples, respectively). Image reconstruction was conducted using Bruker NRecon software (version 2.2.0.6) by applying beam hardening correction.

Fourier-transform infrared spectroscopy (FTIR) was used to identify the functional groups and chemical bonds present at the surface of the samples. The analysis was performed on a spectrometer model 6800FV from JASCO Analitica. The measurements were made by total attenuated total reflectance using the ATR ProOne accessory and making a blank in the air without the need to disperse or treat the sample. For the acquisition of spectra, a standard spectral resolution of 4 cm^–1^ was used in the spectral range of 4000–400 cm^–1^ as well as 64 accumulations per sample.

X-ray photoelectron spectroscopy (XPS) determined the samples’ surface chemistry. The spectra were recorded on a physical electronic spectrometer (PHI Versa Probe II) using monochromatic Al Kα radiation (15 kV, 1486.6 eV), a dual beam charge neutralizer for analyzing the core-level signals of the elements of interest, and a hemispherical multichannel detector. The sample spectra were recorded with a constant pass energy value of 29.35 0.125 eV/step and a beam diameter of 200 μm. The energy scale was calibrated using Cu 2p_3/2_, Ag 3d_5/2_, and Au 4f_7/2_ photoelectron lines at 932.7, 368.2, and 83.95 eV, respectively. Atomic concentration percentages of the characteristic elements were determined by considering the corresponding area sensitivity factor for the different spectral regions measured. The Multipak 9.6 software was used for data acquisition and analysis. A Shirley-type background was subtracted from the signals. Recorded spectra were always fitted using Gaussian–Lorentzian curves to determine the binding energy of the different element core levels more accurately.

The thermal behavior of the samples and precursors was investigated by a thermogravimetric analysis. A thermogravimetric analyzer, Mettler Toledo model TGA/DSC 1, was used in a range from 30 to 900 °C with an airflow of 50 mL/min and heating velocity of 10 °C, with 70 μL of alumina crucible and 10 mg of sample.

The ^29^Si and ^27^Al NMR spectra were recorded using a high-definition nuclear magnetic resonance spectrometer, Bruker model AVIIIIHD 600, narrow bore with a magnetic field of 14.09 T, at 156.4 MHz (Al frequency) and 119.2 MHz (Si frequency) with a 4 mm dual-resonance CPMAS probe using zirconia rotors at slew rates of 13 kHz. Experiments with ^27^Al were performed with proton decoupling (continuous wave sequence) by applying a single pulse (π/18), an excitation pulse of 0.7 μs, and a relaxation delay of 1 s to obtain 1000 scans. Chemical shifts were referenced to an external 1 M Al­(NO_3_)_3_ solution. Experiments with ^29^Si were also performed with proton decoupling (continuous wave sequence) by applying a single pulse (π/2), an excitation pulse of 8.5 μs, and a relaxation delay of 60 s to obtain 1000 scans. Chemical changes were referenced with an external solution of tetramethylsilane (TMS).

The adsorption equilibrium tests were carried out with the CO_2_, CO, and H_2_ gases at 30, 50, and 100 °C, with pressures between 0 and 760 mmHg, using a surface area and porosity analyzer, Micromeritics 3Flex model.

## 3 Results and Discussion

### 3.1 Physico-Chemical Properties and Textural Characterization

The chemical compositions of the geopolymer samples, analyzed through XRF, are presented in Table 2. As expected, the main components of the compositions are silica (SiO_2_), alumina (Al_2_O_3_), sodium oxide (Na_2_O), and iron oxide (Fe_2_O_3_). However, there are considerable amounts of titanium (TiO_2_), calcium (CaO), and phosphorus (P_2_O_5_) oxides in the GP sample. The oxides in the geopolymer samples follow the analysis of precursors (metakaolin and phosphate mining tailings) carried out by ref 22

**Table 2:** Total Oxide Composition and Textural Characterization of the Samples

	G65s	G65	G80s	G80
composition, wt %				
SiO_2_	42.42 ± 0.37	43.53 ± 0.37	41.52 ± 0.37	41.9 ± 0.37
Al_2_O_3_	22.84 ± 0.18	20.92 ± 0.17	22.18 ± 0.18	20.79 ± 0.17
Na_2_O	9.10 ± 0.02	12.48 ± 0.37	9.26 ± 0.32	12.63 ± 0.37
Fe_2_O_3_	7.40 ± 0.09	6.93 ± 0.18	7.27 ± 0.18	6.77 ± 0.18
TiO_2_	3.07 ± 0.05	2.96 ± 0.02	3.06 ± 0.02	2.83 ± 0.02
CaO	1.74 ± 0.03	1.73 ± 0.09	1.82 ± 0.09	1.60 ± 0.09
P_2_O_5_	1.49 ± 0.04	1.53 ± 0.05	1.53 ± 0.05	1.46 ± 0.05
K_2_O	0.38 ± 0.01	0.37 ± 0.03	0.36 ± 0.03	0.36 ± 0.03
MgO	0.36 ± 0.00	0.27 ± 0.03	0.32 ± 0.03	0.25 ± 0.03
MnO	0.33 ± 0.00	0.30 ± 0.01	0.29 ± 0.00	0.29 ± 0.00
CuO	0.04 ± 0.00	0.03 ± 0.00	0.03 ± 0.00	0.03 ± 0.00
Cl	0.02 ± 0.00	0.02 ± 0.00	0.01 ± 0.00	0.01 ± 0.00
ZnO	0.02 ± 0.00	0.01 ± 0.00	0.01 ± 0.00	0.01 ± 0.00
SO_3_				0.01 ± 0.00-
LOI	10.80	8.89	12.31	11.04
textural characterization				
density (g/cm^3^)	2.400 ± 0.001	2.450 ± 0.002	2.400 ± 0.002	2.450 ± 0.002
BET area (m^2^/g)	301	187	337	154
total pore volume (cm^3^/g)	0.131	0.075	0.133	0.053
pore size (Å)	55.89	52.68	61.81	64.25

Regarding the influence of curing temperature, the samples did not present a significant difference in composition. Finally, when comparing the submersed cured samples with those only dry cured, the amounts of silica and aluminum oxides are very similar; however, sodium oxide is considerably higher for both dry-cured samples.

The density, surface area, total pore volume, and pore size of the samples are shown in Table 2. The density of the samples was very similar in the samples with and without a submerged cure. The density of the samples increases as the surface area and total pore volume decreases, which is expected and consistent with the results obtained elsewhere^3^ for zeolite and geopolymer composites.

The surface areas of the submerged samples are significantly larger than those of the nonimmersed curing samples, as are the total pore volumes (Table 2). This enhancement can be attributed to the combined effects of increased zeolitic phase formation and changes in the geopolymerization process during submerged curing.^26^ The increased water availability facilitates precursor dissolution and promotes the hydrothermal synthesis of zeolitic structures, which not only enhances crystallinity but also contributes to a more open and interconnected pore structure. As a result, both the surface area and total pore volume increase significantly.^27^ As shown in Figure 2, the submerged samples exhibit more intense peaks in the XRD analysis, indicating increased crystallinity of zeolitic phases compared to the nonsubmerged samples.

The formation of these zeolitic phases not only increases the surface area and total pore volume but also improves the material’s adsorption capacity. This is because zeolitic structures are known for their high surface area, well-defined pore sizes, and ion-exchange properties, all of which contribute to increased adsorption performance.^27–29^ The submerged samples exhibited higher BET surface area and total pore volume than the nonsubmerged ones, indicating that submerged curing influenced the development of porosity but did not directly affect the pore size distribution. The increased surface area and porosity in the submerged samples can be attributed to the enhanced salt dissolution, as shown by XRF analysis, which facilitates the formation of a more porous structure during the curing process. Meanwhile, the curing temperature did not significantly affect the pore size; however, the sample cured at 65 °C exhibited a pore size larger than that of the sample cured at 80 °C.

Although the BET surface areas of the samples in this study are lower than those of other porous CO_2_ adsorbents reported in the literature, they still show promise for CO_2_ capture. For instance, activated carbon produced from tuff materials ion-exchanged with lithium presents a BET surface area of 155 m^2^/g, similar to the lower range of our samples (154–187 m^2^/g).^9^ Moreover, CALF-20, a MOF material, has a BET surface area of 350 m^2^/g, comparable to the higher BET values of the submerged samples (301–337 m^2^/g).^8^ Despite the relatively lower surface areas, our materials benefit from a simpler, cost-effective synthesis and offer a competitive, sustainable alternative for CO_2_ capture due to their zeolitic phases, ion-exchange properties, and favorable pore structure. It is also important to consider that higher surface areas, especially when associated with significant microporosity, may sometimes lead to lower selectivity or reduced structural stability during adsorption processes. In contrast, the balance of surface area and pore architecture in our materials supports both stability and practical performance. Furthermore, the use of industrial waste as a precursor adds considerable value in terms of environmental impact and production cost, which are critical factors when materials are evaluated for large-scale applications.

Open porosity (void space), closed porosity (pores, trapped bubbles), and total porosity, evaluated by μCT, are presented in Table 3. The sample G65s presented the highest open porosity and G80 the lowest. The volume of closed porosity is quite similar for G65s and G80s, while G65 and G80 had a greater closed porosity. However, in Figure 1, the black spots represent the closed pores. The number of pores in G80 seems to be larger than the others. This can be explained by the nanosize of the pores, which is a size that the equipment is unable to recognize.

**Table 3:** Quantification of Porosity Using Micro-Computed Tomography

sample	open porosity (vol %)	closed porosity (vol %)	total porosity (vol %)
G65s	10.218	2.428	12.450
G65	1.550	4.893	6.367
G80s	1.477	2.300	3.743
G80	0.712	3.645	4.331

**Figure 1:**
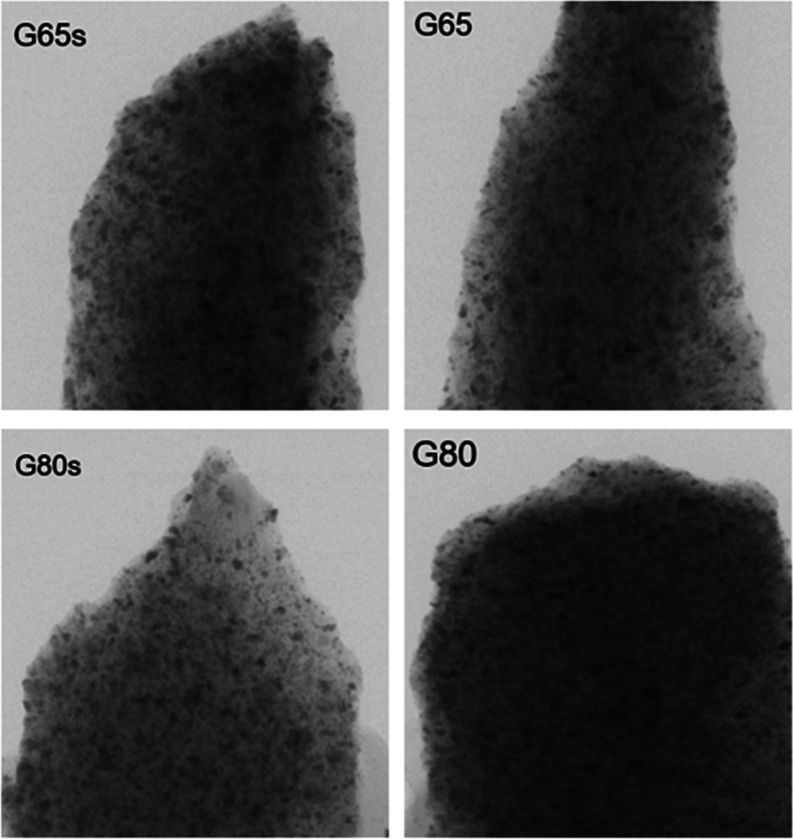
Micro-CT imaging of the geopolymer samples.

### 3.2 Mineralogical Composition, Crystallinity, and Morphology

The XRD patterns of the geopolymer samples are given in Figure 2. The presence of mineralogical phases in the geopolymers is reported in many studies, especially when the synthesis process includes a thermal cure.^30–32^

**Figure 2:**
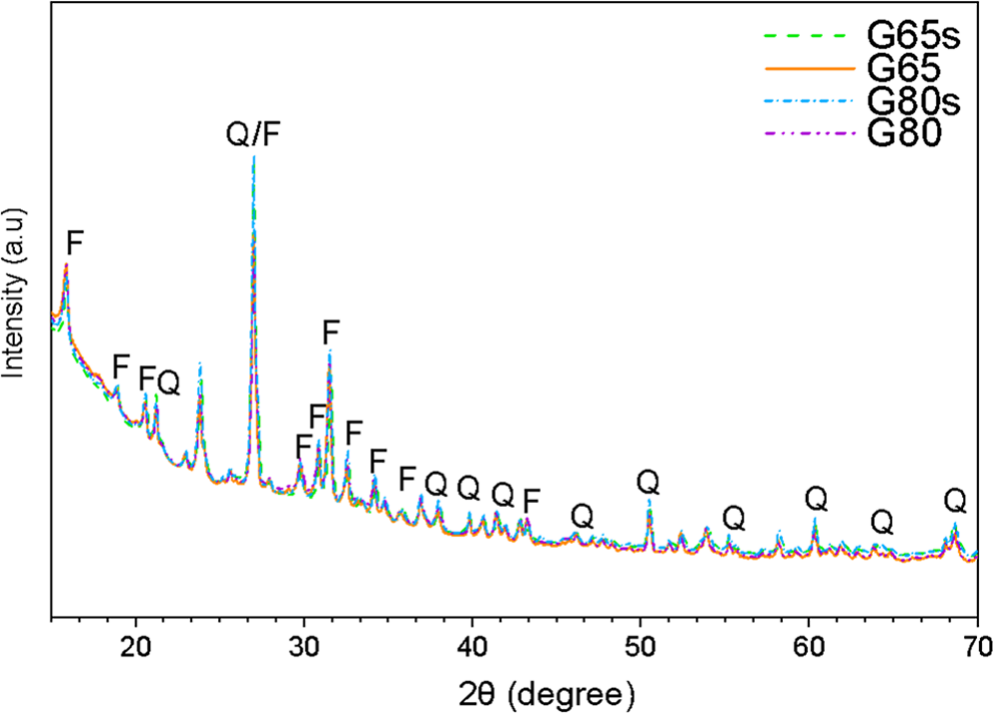
XRD patterns of the geopolymer samples, with peaks labeled as Q for quartz and F for faujasite.

For the precursor materials, the phosphate waste is crystalline, so the XRD patterns showed some crystalline phases, which are mainly composed of quartz (SiO_2_—ICSD-62405) at ∼86% and the remainder by anatase (TiO_2_—COD-9008215), vaterite (CaCO_3_—COD-9007475), magnetite (FeO–Fe_2_O_3_—COD-9002329), and goethite (Fe_4_O_8_—COD-9003077).^22^ MK is mainly amorphous (∼97%); however, two crystalline phases were identified, quartz (SiO_2_—JCPDS-46-1045) and fluorite (CaF_2_—JCPDS-35-0816).^18^

Considering the precursor materials, during curing, different crystalline phases may be formed, even in geopolymers with identical formulations. However, in this case, the crystallographic structures of the four samples are considerably identical. In the diffractograms, a broad hump can be seen in the range from 15 to 40°, indicating the presence of amorphous aluminosilicate phases, which is expected, since the geopolymer is a predominantly amorphous material. Two crystalline phases were formed, alpha quartz (SiO_2_ ICSD 062406) and faujasite (Na_5.12_Al_52.35_Si_139_O_362.88_(OH)_32_H_39.594_ ICSD 024867). In addition, some of the diffraction peaks were sharp and intense, indicating a high degree of crystallinity of these phases.^33^

The morphology of the microstructure of the samples is shown in Figures 3–6. In these figures, the dense and homogeneous structure of the samples can be noticed, confirming the large amount of oligomers (Si–Al) in the reaction medium, which increases the degree of geopolymerization of the material.^34^ Unlike samples G65s and G80s, samples G65 and G80 are less compacted, which may be related to the dry curing, since without submersion, the samples have a high amount of salts, as presented through XRF analysis.

**Figure 3:**
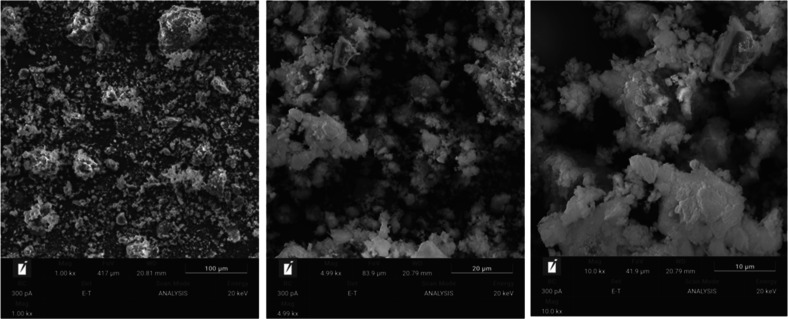
SEM images of the G65s sample.

**4:**
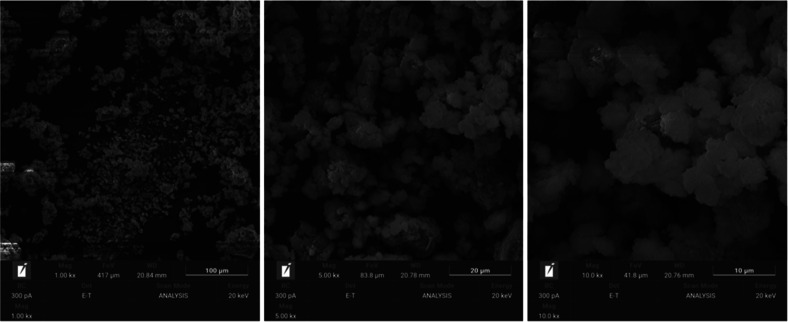
SEM images of the G65 sample.

**5:**
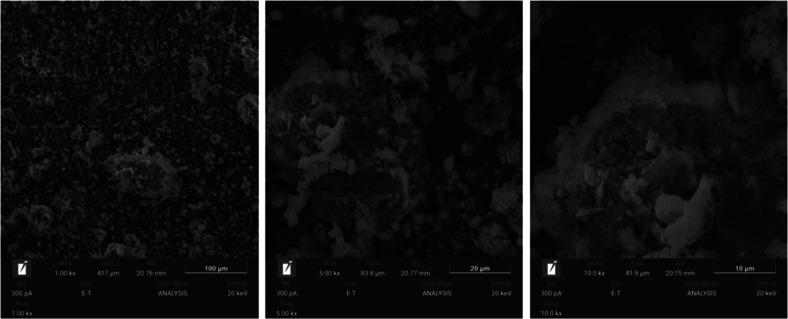
SEM images of the G80s sample.

**Figure 6:**
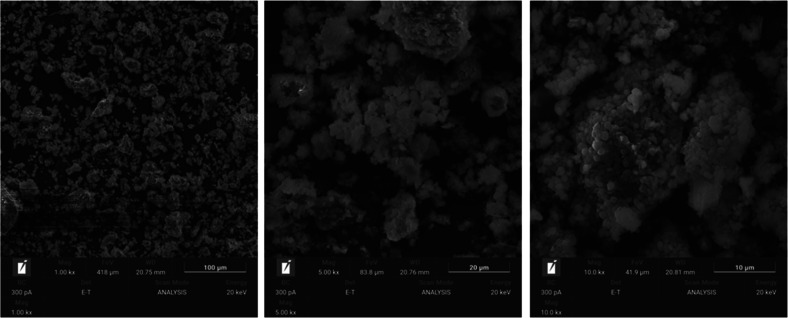
SEM images of the G80 sample.

### 3.3 Chemical Structure

The FTIR-ATR spectra for the samples are shown in Figure 7. The bands between the orange dashed lines, in the region from 4000 to 2600 cm^–1^, are attributed to the stretching vibrations of Si–OH, OH, and Si–OH–Al groups. The band between 1750 and 1650 cm^–1^, red dashed lines, is assigned to the bending vibration of the OH group.^27,32,35^

**Figure 7:**
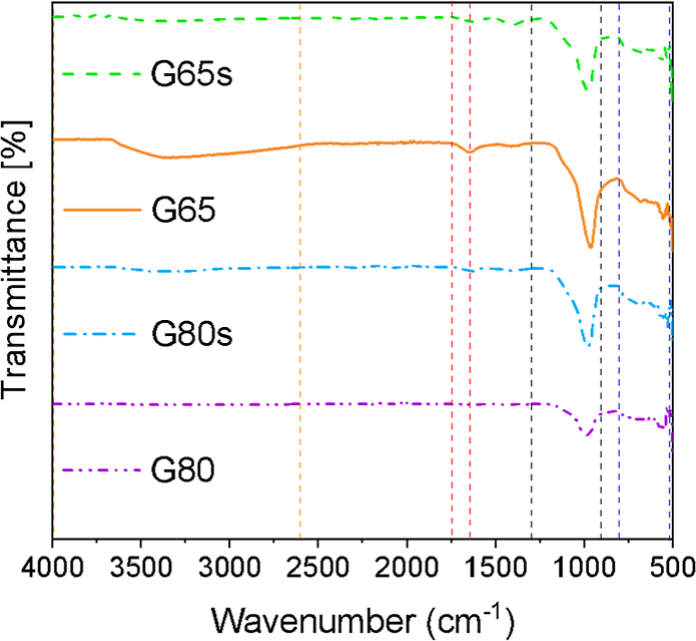
FTIR-ATR spectra of the samples.

The bands in the region ∼1500 to 1400 cm^–1^ represent the formation of carbonates, and in this case, their absence confirms that there are no carbonates in the samples, which is interesting, since the presence of carbonates can compromise the chemical and physical properties of materials, particularly geopolymers^32,36^

The bands between the black dashed lines, in the 1300 to 900 cm^–1^ range, are characteristic of aluminosilicate materials, which correspond to the asymmetric stretching vibration of Si–O–T-type structures, where T is Si or Al.^35^ The peak at ∼1070 cm^–1^ represents the symmetric stretching vibration of the Si–O bonds and is identified in all sample spectra. The bands from 800 to 500 cm^–1^, at the blue dashed lines, are characteristic of symmetrical Si–O–Si group vibrations, and the bands between 730 and 554 cm^–1^ correspond to the vibrational bands of zeolitic materials, in this case, faujasite, zeolite 13X type.^37^

### 3.4 Surface Chemistry

XPS analysis was performed to know the chemical states and atomic composition of the surface of the samples particles. The identification of the peaks was based mainly on the *Handbook of X-ray Photoelectron Spectroscopy*.^38^ Overall, the sample spectra are very similar, changing mostly the intensity of the peaks. The area of the peaks, atomic concentrations, and weight, obtained with the MultiPak software, are reported in Table S1. As expected, the values are very similar for the submersed samples and the nonsubmersed samples.

The XPS spectra of the samples are shown in Figure 8 and the decomposed high-resolution core-level spectra are presented in Figures S1–S4. The XPS quantification, atomic concentration, and weight of the samples are presented in Table 4, and the XPS quantification and area of the deconvoluted peaks of the samples are presented in Table S1; as expected, the values are very similar for the submersed samples and the not submersed samples.

**Figure 8:**
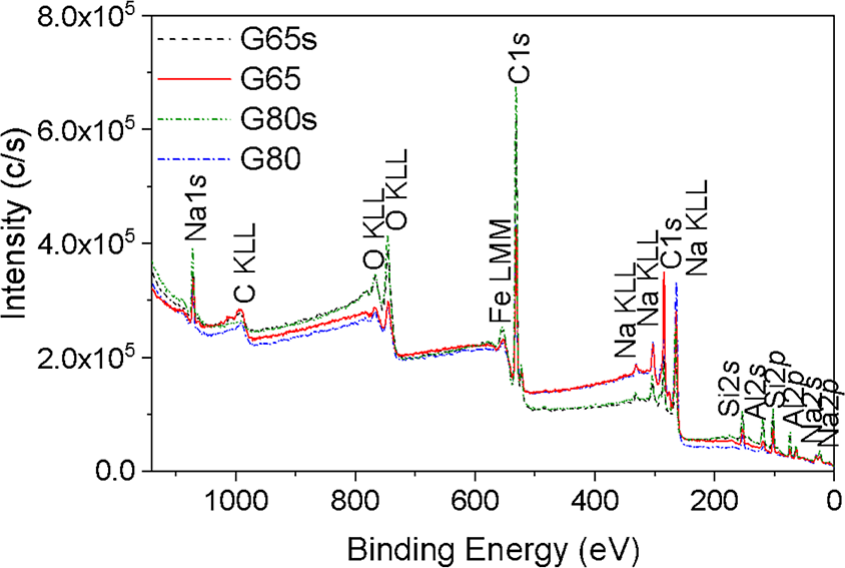
XPS survey spectra of the geopolymer samples.

**Table 4:** XPS Quantification by Atomic and Weight Concentration of the Samples

	G65s		G65		G80s		G80	
	atomic (%)	weight (%)	atomic (%)	weight (%)	atomic (%)	weight (%)	atomic (%)	weight (%)
C 1s	18.02	11.59	45.35	33.08	17.55	11.27	44.69	33.06
O 1s	50.66	43.41	33.00	32.06	50.63	43.29	35.30	34.79
Na 1s	6.66	8.21	5.71	7.98	7.17	8.81	6.91	9.79
Si 2p	13.07	19.66	10.79	18.41	12.89	19.35	8.48	14.68
Al 2p	11.26	16.28	5.11	8.38	11.54	16.65	4.62	7.68
Fe 2p3	0.16	0.48			0.15	0.45		

For all samples, the positions of the peaks are quite similar; in the deconvolution spectra, the high-resolution C 1s core-level spectra were deconvoluted into four contributions, the contribution at 284.8 eV is correspondent to adventitious carbon (contamination), C–C, and/or C=C groups, at 286.3–287.0 eV, it is related to the C–OH and C–O–C groups, the contribution at 288.4–289.4 eV is characteristic of the carbonate group, this group can also be assigned to the COOH or OH–C–OH bonds,^39^ and the peak at 290.7 −292.0 eV is correspondent to π → π* satellite shakeup.

The O 1s spectra were deconvoluted in three contributions: that at 529.9–530.5 eV corresponds to lattice oxygen of the Na_2_O group; the contribution at 531.5 eV corresponds to O–H, which can be assigned to water and/or the carbonate^–^ group; and the peak at 532.8–532.9 eV is related to Si–O groups.

The high-resolution Na 1s spectra present a single peak at 1071.6–1072.4 eV, which is characteristic of Na^+^ in sodium aluminosilicate. Moreover, the Si 2p and Al 2p spectra have a single peak at 102.4–102.6 and 74.2–74.4 eV, respectively, and these peaks are also characteristic of aluminosilicate. The submersed samples presented a small amount of iron; the peaks are at 711.0 and 711.1 eV, for G80s and G65s, respectively, and are assigned to the presence of Fe^3+^.

### 3.5 Thermal Behavior

The thermal behavior of the geopolymer samples is presented in Figure 9, which shows the thermogravimetric degradation and DTG curves. A significant decrease in the weight loss curves of the samples can be seen from room temperature to approximately 250 °C. The samples presented a mass loss of 13.85, 14.57, 14.83, and 13.03%, with the peaks at 123.5, 104.7, 125.6, and 109.1 °C, for the G65s, G65, G80s, and G80 samples, respectively. This gradual decrease in mass is related to the elimination of free and interstitial water in the sample.^40–42^ Moreover, the stable mass loss from 250 °C to approximately 680 °C can be explained by the release of chemically bound water and hydroxyl (OH) groups by dehydroxylation.^41–44^

**Figure 9:**
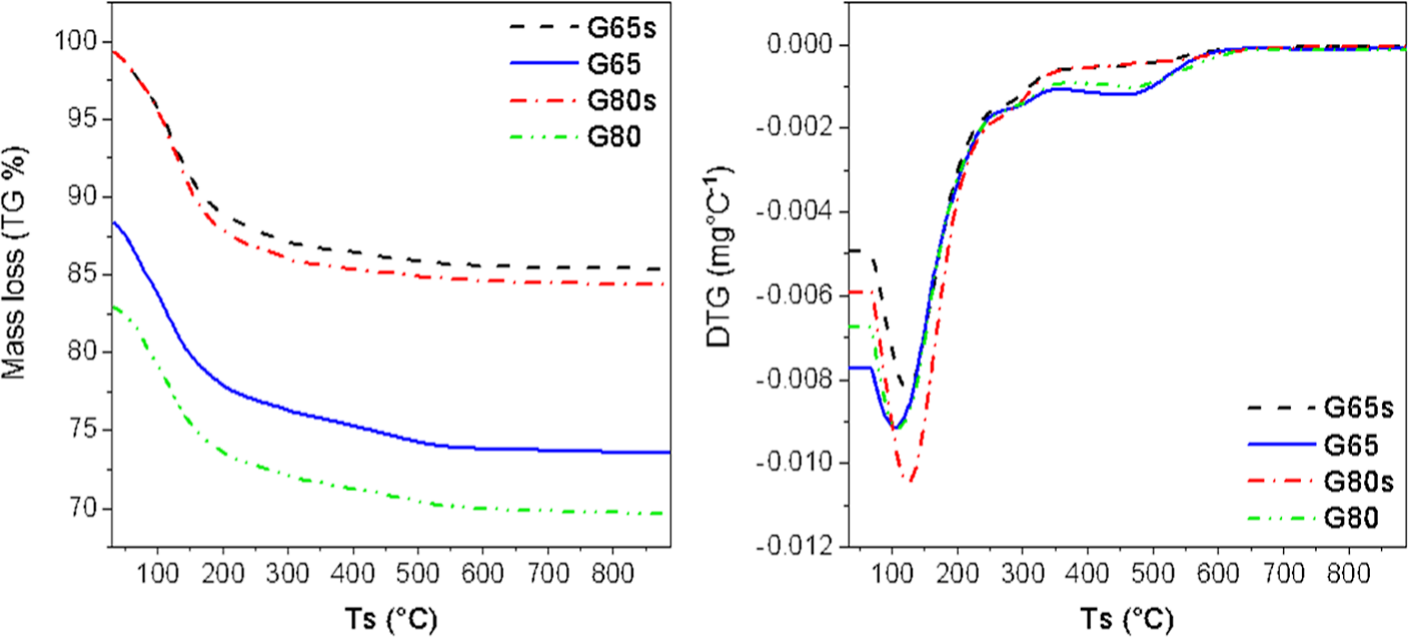
TG-DTG curves of geopolymer samples.

Comparing the endothermic peaks with the porosity analysis, the two submersed samples (G65s and G80s) presented higher total pore volume than the nonsubmersed ones. This means that more space is available, and consequently, the interstitial water trapped in the pores requires a higher temperature to be removed.^40^ This increase in the temperature of the endothermic peak indicates a stronger binding between the water and the network in the geopolymer gel phase.^40,44^

Figure 10 shows the DSC thermograms of the samples. The samples presented small endothermic peaks located at 118.9, 124.1, 130.2, and 82.4 °C, for the G65s, G65, G80s, and G80 samples, respectively. This confirms the relationship of the peaks to the removal of free and interstitial water contained in the geopolymers.^40,45^

**Figure 10:**
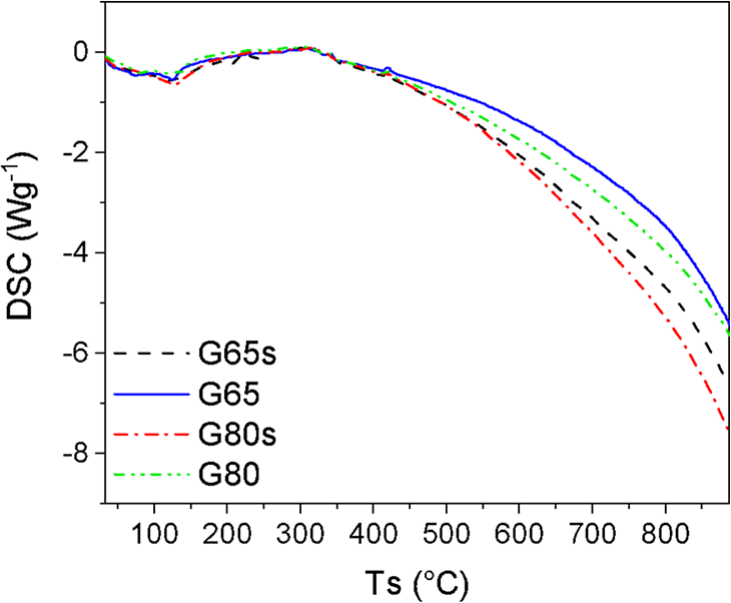
DSC thermogram curves of geopolymer samples.

### 3.6 Coordination of Si and Al

Solid-state NMR spectroscopy is an important analysis for investigating the characteristics of the geopolymeric materials because it provides data regarding the coordination state of alumina units in the structure of precursor materials and geopolymers and consequently their reactivity.^18^

Figure 11 shows the ^27^Al and ^29^Si NMR spectra of all the geopolymer samples, since they are very similar, and only the intensity of the peaks change. It can be observed that the ^27^Al spectra present one sharp peak at ∼61 ppm, which corresponds to Al­(IV), indicating the presence of tetrahedral aluminum in the sample, and two smaller peaks at −10 ppm and one at −21 ppm, which corresponds to Al­(VI), octahedral aluminum. This may be related to zeolite,^46^ since in the XRD analysis, the samples presented some crystalline peaks, which were related to faujasite. The absence of octahedral and penta-coordinated aluminum sharp peaks confirms that the geopolymerization reaction is complete.^47–50^

**Figure 11:**
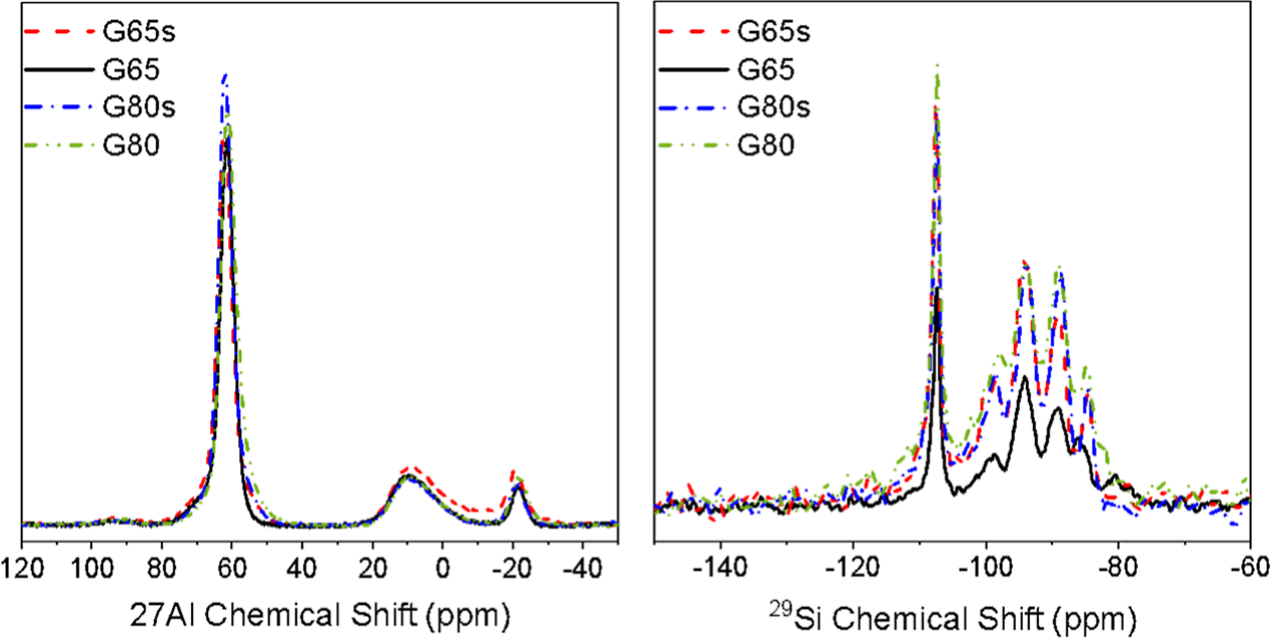
^27^Al and ^29^Si NMR spectra of geopolymer samples.

The ^29^Si NMR spectra presents five significant peaks; since they presented the same peaks, the G65s sample was chosen for deconvolution, which is presented in Figure 12. The most intense peak at −107 ppm corresponds to the Q^4^0Al silicon types. The peaks at approximately −99, −100, and −102 ppm correspond to the Q^4^1Al unit. The peaks at −94, −89, and −85 ppm correspond to the Q^4^2Al, Q^4^3Al, and Q^4^4Al units, respectively.^51,52^ As expected, the presence of peaks at approximately −107 ppm confirms the formation of aluminosilicate gels, alternating between silica and alumina tetrahedrons. Also, the presence of a peak near −110 ppm can be assimilated to the presence of the unreacted crystalline phase of the precursor materials and the use of sodium.^53^

**Figure 12:**
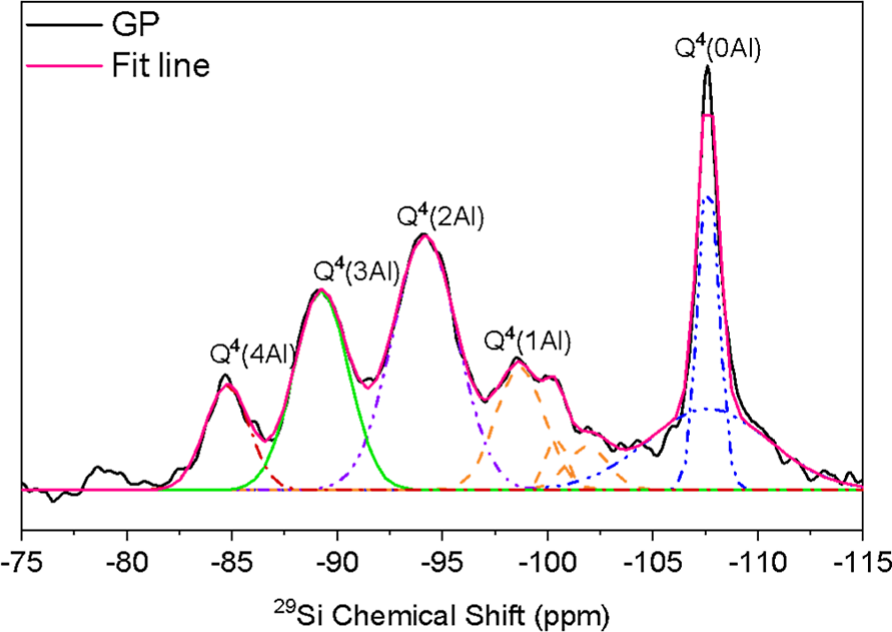
Deconvolution of the ^29^Si NMR spectra of the G65s sample.

### 3.7 Adsorption–Desorption Equilibrium Isotherms

The adsorption capacity depends on many factors, including surface area, volume, density, pore size and volume, and connectivity between the pores.^54^ Since the physicochemical characteristics of the geopolymers synthesized in this study are very similar for both types of curing (dry or submersed), the adsorption/desorption capacities of the samples for all gases are expected to be similar.

The CO_2_, CO, and H_2_ adsorption equilibrium isotherms were obtained at three different temperatures, 30, 50, and 100 °C, and pressure range of 0 and 760 mmHg. To analyze the experimental data, different mathematical models were applied (Langmuir, Freundlich, Sips, and Redlich Peterson). Among these, the Sips model provided the best fit to the experimental isotherms.

The Sips model combines the well-known Langmuir and Freundlich models designed to account for the heterogeneity of adsorptive systems. Its parameters are primarily influenced by operating conditions such as pH, temperature, and solute concentration.^55^ Due to its high accuracy, it is the most widely applied model.^28^ In gas adsorption, several factors influence the adsorption behavior. Temperature affects the strength of interactions between gas molecules and the adsorbent surface, while pressure impacts the adsorption capacity and gas distribution within the porous structure. Additionally, surface chemistry and textural properties, such as pore size and distribution, play a key role in determining adsorption efficiency.^55^

Figures 13–15 show the isotherms with the respective suitable model. The CO_2_ adsorption isotherms at the three temperatures studied for all the samples presented favorable shapes, since the CO and H_2_ isotherms are quite linear.^56^ The parameters obtained by applying the Sips model are presented in Table 7 at the end of the section. Table S2 presents the data obtained by applying the Langmuir, Freundlich, and Redlich Peterson, along with the Sips model.

**Figure 13:**
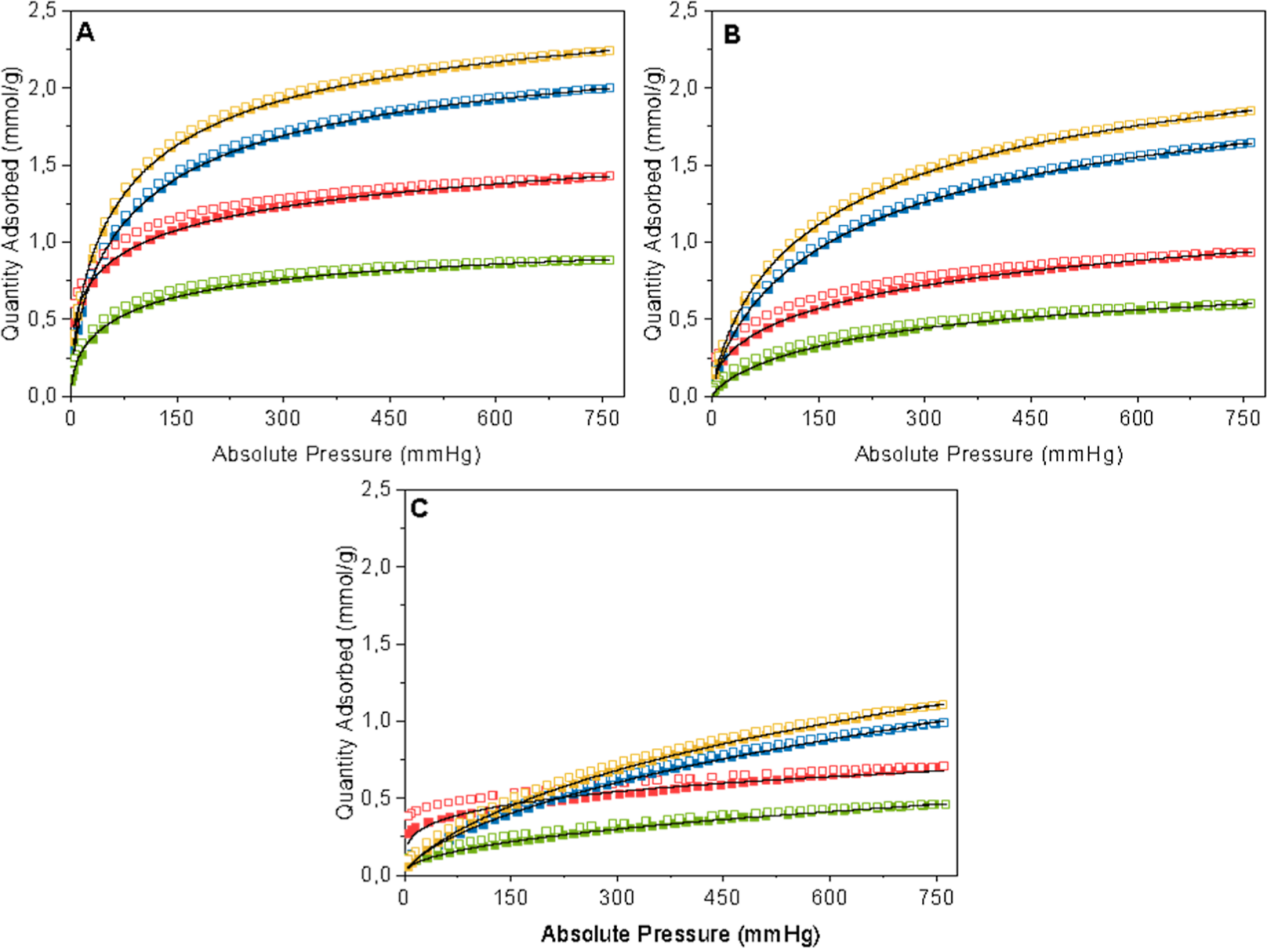
Adsorption (closed squares) and desorption (open squares) isotherms of CO_2_ on G65s (blue), G65 (red), G80s (yellow), and G80 (green) at 30 °C (A), 50 °C (B), and 100 °C (C). The black line indicates the Sips isotherm model.

**Table 5:** Comparison of the Adsorption Capacity of the Samples with Geopolymers Reported in the Literature at 30–35 °C and 1 bar^a^

	precursor materials			
adsorbent	aluminosilicate source	alkaline activator	CO_2_ adsorption (mmol/g)	reference
G10	Metakaolin, fumed silica	Potassium silicate	0.62	^25^
G13			0.58	
G23			0.57	
K-G_2_	Metakaolin	NaOH, potassium disilicate	0.58	^7^
Na-G_1,2_			1.95	
GEO	Metakaolin, silica fume	Potassium hydroxide	0.19	^66^
MCR-1	Metakaolin, calcined rice husk ash	NaOH	0.80	^18^
MF-1	Metakaolin, fly ash		0.78	
MR-1	Metakaolin, rice husk ash		0.69	
MFCR-1	Metakaolin, fly ash, calcined rice husk ash		0.63	
MFR-1	Metakaolin, rice husk ash, fly ash	Sodium silicate	0.68	
MF-2	Metakaolin, fly ash		0.64	
MPW	Metakaolin/Phosphate mining tailing	NaOH, sodium silicate	1.9	^22^
TEPA-WPGS3	Fly ash	NaOH, sodium silicate	1.97	^67^
G65s	Metakaolin/Phosphate mining tailing	NaOH, sodium silicate	2.00	this work
G65			1.42	
G80s			2.24	
G80			0.88	

a Pressure: 1 bar = ∼750 mmHg = ∼1 atm.

**Figure 14:**
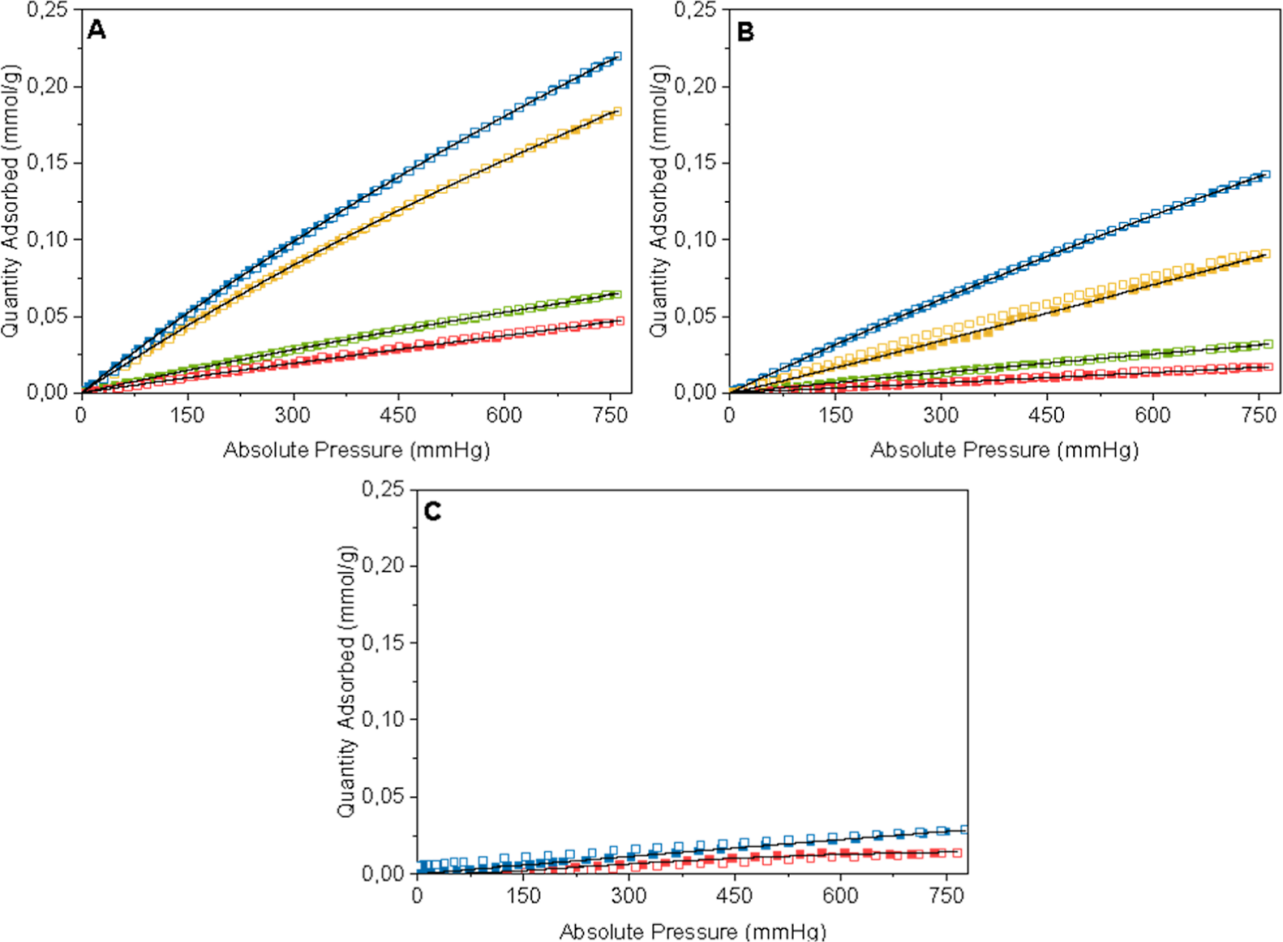
Adsorption (closed squares) and desorption (open squares) isotherms of CO on G65s (blue), G65 (red), G80s (yellow), and G80 (green), at 30 °C (A), 50 °C (B), and 100 °C (C). The black line indicates the Sips isotherm model.

**Figure 15:**
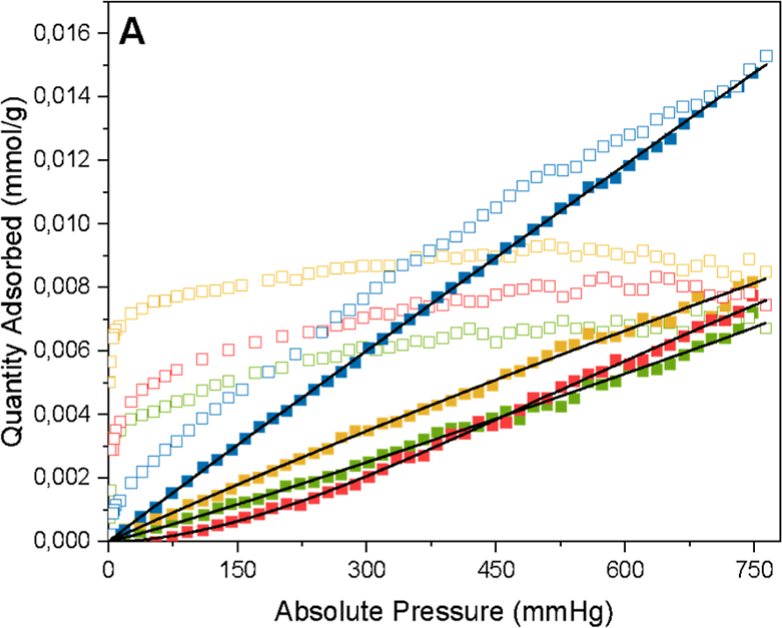
Adsorption (closed squares) and desorption (open squares) isotherms of H_2_ on G65s (blue), G65 (red), G80s (yellow), and G80 (green), at 30 °C, and the black line indicates the Sips isotherm model.

**Table 6:** CO_2_, CO, and H_2_ Gas Adsorption Capacity and Selectivity at 30 °C and 1 bar

	gas adsorption capacity (mmol/g)			selectivity	
sample	CO_2_	CO	H_2_	CO_2_/CO	CO_2_/H_2_
G65s	2.00	0.22	0.015	9.09	133.33
G65	1.42	0.05	0.007	28.4	202,86
G80s	2.24	0.18	0.008	12.44	280.00
G80	0.88	0.06	0.007	14.66	125.71

**Table 7:** Sips Model Parameters Applied to the Sample’s Equilibrium Data

			Sips model			
gas	temp	sample	*K*_S_ (mmHg)	*q* (mmol/g)	*n*_S_	*R* ^2^
CO_2_	30 °C	G65s	0.043	2.517	0.676	0.999
		G65	0.131	2.307	0.379	0.999
		G80s	0.048	2.758	0.682	0.999
		G80	0.065	1.159	0.590	0.999
	50 °C	G65s	0.015	2.325	0.761	0.999
		G65	0.034	1.855	0.515	0.999
		G80s	0.017	2.525	0.767	0.999
		G80	0.011	0.890	0.792	0.999
	100 °C	G65s	0.003	4.872	0.652	0.999
		G65	2.167 × 10^–4^	6.394	0.269	0.990
		G80s	0.004	3.125	0.734	0.999
		G80	6.596 × 10^–5^	3.220	0.464	0.997
CO	30 °C	G65s	3.227 × 10^–4^	1.226	0.982	0.999
		G65	7.473 × 10^–5^	1.048	0.970	0.999
		G80s	3.540 × 10^–4^	0.887	0.996	0.999
		G80	2.580 × 10^–4^	0.418	0.989	0.999
	50 °C	G65s	1.867 × 10^–4^	1.200	0.992	0.999
		G65	4.144 × 10^–6^	5.166	1.007	0.999
		G80s	1.5523 × 10^–6^	5.635	1.049	0.999
		G80	1.558 × 10^–4^	0.314	0.991	0.999
	100 °C	G65s	9.811 × 10^–5^	0.338	1.029	0.999
		G65	5.379 × 10^–6^	0.018	2.022	0.987
H_2_	30 °C	G65s	7.633 × 10^–6^	2.894	0.983	0.997
		G65	4.357 × 10^–6^	0.020	1.784	0.997
		G80s	1.457 × 10^–4^	0.091	0.984	0.997
		G80	8.071 × 10^–7^	6.402	1.083	0.996

gas	sample	–Δ*H* (KJ·mol^–1^)
CO_2_	G65s	–9.538
	G65	–9.050
	G80s	–9.543
	G80	–8.213
CO	G65s	–27.347
	G65	–20.475
	G80s	–27.751
	G80	–28.200

Figure 13 presents the CO_2_ adsorption isotherms carried out at 30 (A), 50 (B) and 100 °C (C). At 30 °C, the submersed samples presented a higher adsorption capacity than the nonsubmersed ones, reaching 2.25, 2.00, 1.43, and 0.88 mmol/g, for samples G80s, G65s, G65 and G80, respectively. The same happens for adsorption at 50 °C; the highest gas adsorption, of 1.85 mmol/g, was obtained with sample G80s, followed by sample G65s, with an adsorption of 1.64 mmol/g. Samples G65 and G80 had adsorption capacities of 0.93 and 0.60 mmol/g, respectively.

The observed adsorption performance can be attributed to the surface properties and porosity of the geopolymers, as confirmed by characterization techniques such as XRF, SEM, and BET surface area analysis. A larger surface area provides more active sites for adsorption, while optimal pore volume and connectivity facilitate efficient diffusion of CO_2_ molecules into the material. Additionally, the total pore volume and surface area influence the overall capacity and structural stability, which are crucial for enhancing the CO_2_ uptake by providing suitable adsorption sites.

A limitation of geopolymeric materials, such as zeolites, is that their adsorption capacity decreases with increasing temperature, as is well-documented in the literature. Although CO_2_ adsorption occurs more rapidly at higher temperatures due to enhanced kinetic processes, the amount of CO_2_ adsorbed at equilibrium decreases because of thermodynamic limitations.^8^ This behavior further supports the idea that CO_2_ capture occurs through physical adsorption, primarily involving the microporous structure and surface area.^22,57–59^ Thus, as expected, the adsorption at 100 °C presented the same pattern as that at 30 °C, but in a very small, adsorbed amount. Once again, the highest adsorption of 1.11 mmol/g was achieved with the G80s sample, followed by G65s, with an adsorption of 0.99 mmol/g, while the G65 and G80 samples had adsorption capacities of 0.70 and 0.46 mmol/g, respectively.

In the adsorption process, two types of adsorption can occur: chemisorption, which involves weak interactions and chemical bond formation between the adsorbent and adsorbate, typically limited to the first surface layer, and physisorption, which involves stronger interactions, such as van der Waals and electrostatic forces, without altering the nature of the species.^6^ Consistent with the structural properties of the samples, the adsorption process in this study was exclusively physical (physisorption), as indicated by the isosteric heat values.^8,9^ These values align with those typically observed in physisorption processes, which involve weaker interactions between the adsorbate (CO_2_) and adsorbent, characteristic of van der Waals forces. This allows for easy regeneration of the material without structural modifications, confirming the physisorption mechanism on the geopolymers.

The isosteric heat of adsorption is a crucial parameter in understanding the adsorption process, as it reflects the strength of molecular–scale interactions between the adsorbate and adsorbent. Generally, values below 80 kJ/mol are indicative of physisorption, whereas values in the range of 90–100 kJ/mol suggest stronger chemisorption.^8,9^ In this study, the isosteric heat of adsorption was determined using the Clausius–Clapeyron equation.^60,61^ By plotting ln *P* versus 1/*T*, the isosteric heat of adsorption was obtained from the slope of the linear fit. While no literature data on the isosteric heat for CO_2_ adsorption on the geopolymer surface were found, zeolitic materials were used as a reference for comparison.

Other studies have found the value of the isosteric heat for CO_2_ adsorption in the zeolite 4A type to be 47.8 kJ·mol^–1^.^61^ In zeolite 4A it is ∼43 kJ·mol^–1^,^62^ in zeolite NaX and NaY types it is 53.8 and 40.8 kJ·mol^–1^, respectively,^62^ and is about 37.20, 31.93, 30.19, and 25.08 kJ·mol^–1^ in zeolites 13X, 5A, 4A, and beta types, respectively.^28^ The isosteric heats of adsorption for CO_2_ obtained in this study with the synthesized geopolymers were 38.49, 38.57, 30.34, and 38.76 kJ·mol^–1^ for the G65, G65s, G80, and G80s samples. Comparing these values with those from zeolites in the literature, it can be concluded that the values in this study are slightly lower for most samples but still closely aligned with those reported for zeolitic materials.

The physisorption mechanism observed in this study, characterized by weak interactions between CO_2_ and the adsorbent (38.49–38.76 kJ/mol), offers practical benefits for CO_2_ capture. The low binding strength allows adsorption–desorption reversibility and reduces the regeneration energy requirements, as it does not require high temperatures for desorption. This energy efficiency makes the materials suitable for industrial CO_2_ capture applications, where continuous cycles and sustainability are essential.^8,9^

Table 5 presents the CO_2_ adsorption capacities of the geopolymers reported in the literature and the respective precursor materials. No studies were found that analyze the adsorption capacity at the same temperatures as those analyzed in this study. The geopolymers synthesized presented an adsorption capacity higher than most of the geopolymeric materials reported in the literature, and very close to others. Moreover, there are a wide range of materials reported in the literature that are used for CO_2_ adsorption, such as N-doped ordered mesoporous carbon, which reached an adsorption capacity of 1.4 mmol/g;^59^ coconut shell activated carbon with a maximum adsorption capacity of 3.61 mmol/g;^63^ polyethylenimine-modified porous wood ceramics, which reached an adsorption capacity of 3.06 mmol/g;^64^ and a foam geopolymer-zeolite X composite that presented a very high CO_2_ adsorption capacity of 6.09 mmol/g.^65^ In terms of adsorption capacity, while the G80S geopolymer exhibits moderate CO_2_ uptake of 2.24 mmol/g at 30 °C and 1 bar—lower than the state-of-the-art zeolitic sorbents such as 13X (∼5 mmol/g), its potential advantages lie in factors like cost-effectiveness, thermal stability, and resistance to moisture. These characteristics could make it a viable alternative in specific applications where conventional sorbents face limitations. Further optimization of the material’s composition and surface properties could enhance its performance for targeted CO_2_ capture scenarios.

Figure 14 shows the CO adsorption isotherms carried out at 30 °C (A), 50° (B), and 100 °C (C). The CO adsorption at 30 °C followed the same pattern as the CO_2_ adsorption, but at a very low adsorption capacity. Samples G65s and G80s had an adsorption capacity of 0.22 and 0.18 mmol/g, and samples G65 and G80 absorbed 0.05 and 0.06 mmol/g, respectively. However, for the CO adsorption at 50 °C, the samples adsorption did not have the same pattern, and the submersed samples had higher adsorption. In this case, sample G65s presented the highest adsorption, of 0.14 mmol/g, followed by sample G80s with an adsorption capacity of 0.09 mmol/g. Samples G65 and G80 had an adsorption capacity of 0.02 and 0.03 mmol/g, respectively. For the CO adsorption at 100 °C, samples G80 and G80s did not have significant adsorption (below 0.001 mmol/g). Samples G65s and G65 had a bit higher adsorption, but still very low, at a capacity of 0.03 and 0.01 mmol/g, respectively.

Finally, Figure 15 presents the H_2_ adsorption capacity at 30 °C; for this gas, all samples had a very low adsorption capacity, and the adsorbed amount was 0.015 mmol/g for sample G65s, 0.008 mmol/g for sample G80s, and 0.007 mmol/g for samples G65 and G80. For adsorption capacity at 50 and 100 °C for H_2_ gas, the samples adsorbed an insignificant amount, all less than 0.006 mmol/g.

No studies were found that analyze the adsorption capacity of geopolymers for CO and H_2_. However, a simple comparison with other solids indicates that the values are significantly higher than those obtained in this study with geopolymers. Some studies analyze the adsorption capacity of these gases using zeolites at different temperatures and pressures. Cui et al. analyzed the CO adsorption capacity in zeolites at 25 °C with a pressure of approximately 750 mmHg, obtaining 1.13 mmol/g and 1.82 mmol/g for the NaX and CaA zeolites, respectively.^68^ Cui et al. analyzed the adsorption capacity of LiX, NaX, and KX zeolites, having an adsorption capacity of 0.97, 1.13, and 0.95 mmol/g, respectively, at 25 °C and pressure of 750 mmHg.^69^ Kim and Kim tested zeolites at 25 °C and a pressure of approximately 750 mmH for CO and H_2_ with NaY and NaX zeolites; the NaY zeolite showed an adsorption capacity of 1.10 mmol/g for CO and 0.02 mmol/g for H_2_, and the NaX zeolite had an adsorption of 1.12 and 0.02 mmol/g for CO and H_2_, respectively.^62^ Shrotri et al. tested four different zeolites for H_2_ adsorption at 30 °C and 7500 mmHg, and as a result, the zeolites presented an adsorption capacity of 0.16, 0.21, 0.25, and 0.33 mmol/g for the Na-X, NH_4_-X, Li-X, and Li–H-X zeolites, respectively.^70^ Streb and Mazzotti tested the 13X zeolite for H_2_ adsorption, obtaining an adsorption capacity of 0.22 mmol/g, at 25 °C and 7500 mmHg.^71^

Table 6 presents the adsorption values for CO_2_, CO, and H_2_, along with the CO_2_/CO and CO_2_/H_2_ selectivities at 30 °C and 1 bar for samples G65s, G65, G80s, and G80. The data indicate that the selectivity for CO_2_/H_2_ is higher for the G80s, while G65 shows higher selectivity for CO_2_/CO.

Finally, a brief comparison with MOFs and activated carbon highlights the influence of surface area and other key factors on the adsorption capacity. Despite similar surface areas, Sips isotherm analysis shows that sample G80s achieves a higher CO_2_ adsorption capacity (2.76 mmol/g, at 30 °C) than CALF-20 (2.55 mmol/g, at 28 °C)^8^. Additionally, although sample G80 has a comparable surface area to the lithium-ion-exchanged tuff, it showed a higher adsorption capacity (1.890 mmol/g, at 25 °C)^9^ compared to G80 (1.159 mmol/g, at 30 °C). This suggests that factors beyond the surface area, such as pore structure and ion-exchange properties, play a crucial role in adsorption performance. Furthermore, the materials in this study offer simpler and more cost-effective synthesis, making them a viable and sustainable alternative for CO_2_ capture.

## 4 Conclusions

In this study, metakaolin-phosphate waste-based geopolymer samples with identical compositions were synthesized. However, part of the material was cured at 65 °C and another part at 80 °C. Additionally, some samples were subjected to a submerged curing process for 28 days. The results indicate that the CO_2_ adsorption capacity was influenced by curing conditions, with higher adsorption observed for the sample cured at 80 °C. The CO_2_ adsorption capacity obtained through the isotherms reached 2.24, 2.00, 1.42, and 0.88 mmol/g at 30 °C, for samples G80s, G65s, G65, and G80, respectively, and this capacity decreased with increasing temperature.

The result obtained with sample G80s represents a significant adsorption capacity compared to results obtained in the literature and is a promising material considering its easy preparation with low-cost materials.

For CO and H_2_ adsorption, the samples presented a very low adsorption capacity. This is good and expected since the objective is to use the material for CO_2_ capture. These findings emphasize the potential of metakaolin-phosphate waste-based geopolymers in CO_2_ capture technology, particularly in enhancing the adsorption efficiency through controlling the surface area and pore size.

In addition to their potential for CO_2_ separation, these materials exhibit characteristics that make them promising candidates for integration into blue hydrogen production systems, where hydrogen is generated from natural gas alongside carbon dioxide sequestration. Future research should focus on evaluating the regeneration capability and long-term performance of these geopolymers over multiple adsorption–desorption cycles as these aspects are essential for real-world implementation in gas separation technologies.

## Supplementary Material



## Notes

Decomposed high-resolution core-level XPS spectra corresponding to the samples; detailed XPS quantification results, including atomic concentrations, weights, and deconvoluted peak areas obtained using the MultiPak software; and isotherm fitting data derived from applying the Langmuir, Freundlich, Redlich–Peterson, and Sips models (PDF)
