# Supplementary material for: Influence of Curing Temperature on the Synthesis of a Phosphate-Waste-Based Geopolymer for CO2 Capture and Separation
Source: ACS Appl Energy Mater. 2025 Jun 3;8(12):8004–18. doi: 10.1021/acsaem.5c00426 (PMC12188614; doi:10.1021/acsaem.5c00426)
Supplement: Supplementary file 1 [file ae5c00426_si_001.pdf]

Influence of Curing Temperature on the Synthesis of a Phosphate-Waste Based Geopolymer for CO_2 Capture and Separation

Supporting Information

Mariana Schneider[1,2], Denise Gomes da Silva Costa[1], Enrique Rodríguez-Castellón[2], M. Olga Guerrero-Pérez[3], Dachamir Hotza[1], Agenor De Noni Jr[1], Regina de F. P. M. Moreira[1]*

[1] Department of Chemical Engineering and Food Engineering, Federal University of Santa Catarina, 88040-900 Florianópolis, SC, Brazil

[2] Department of Inorganic Chemistry Faculty of Sciences, E29071 University of Málaga, Málaga, Spain

[3] Department of Chemical Engineering, Faculty of Sciences, E29071 University of Málaga, Málaga, Spain

Corresponding author: Regina Moreira (regina.moreira@ufsc.br)

Surface chemistry

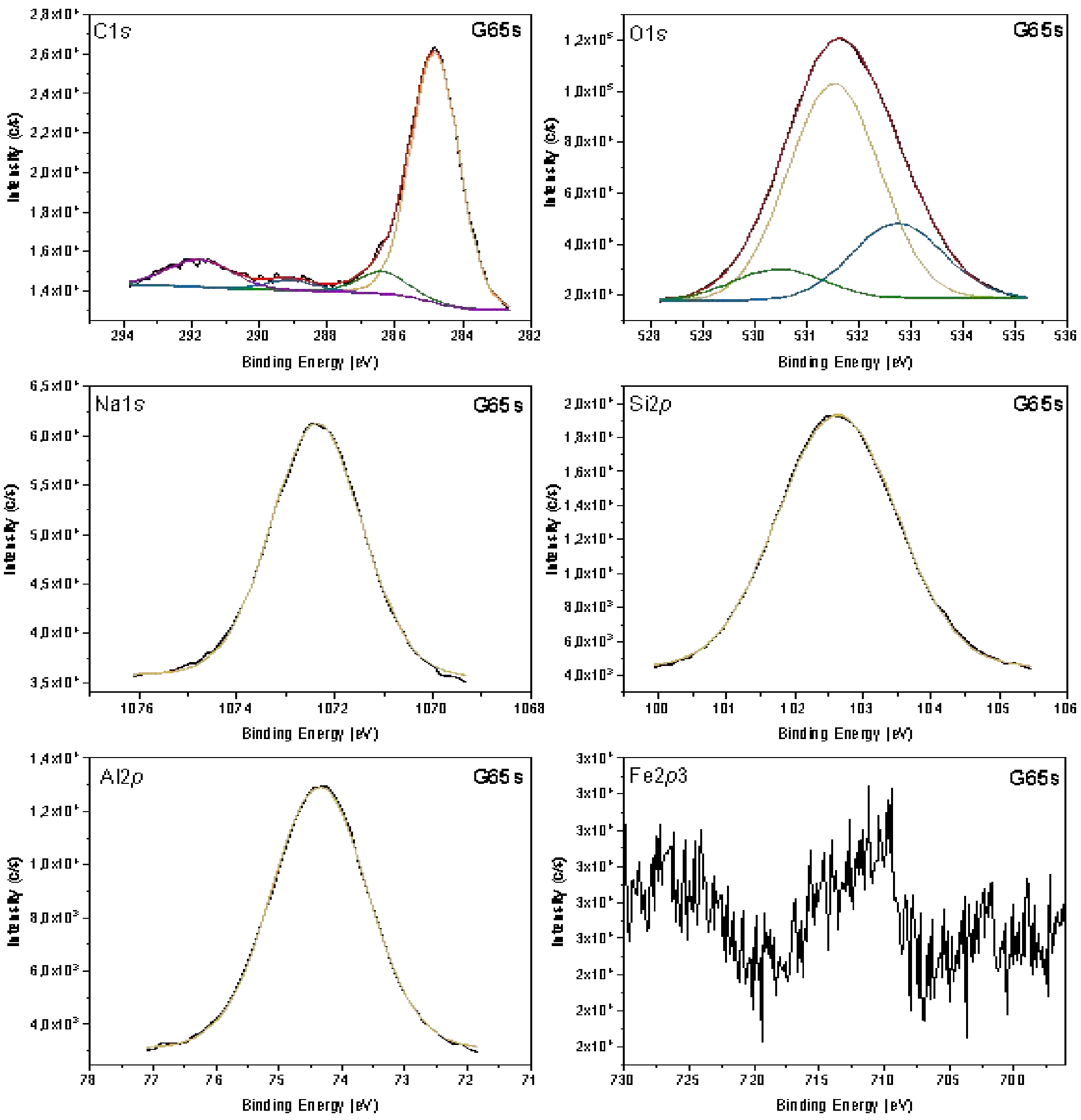

Figure S1. XPS decomposed atom electron orbitals spectra of the G65s sample.

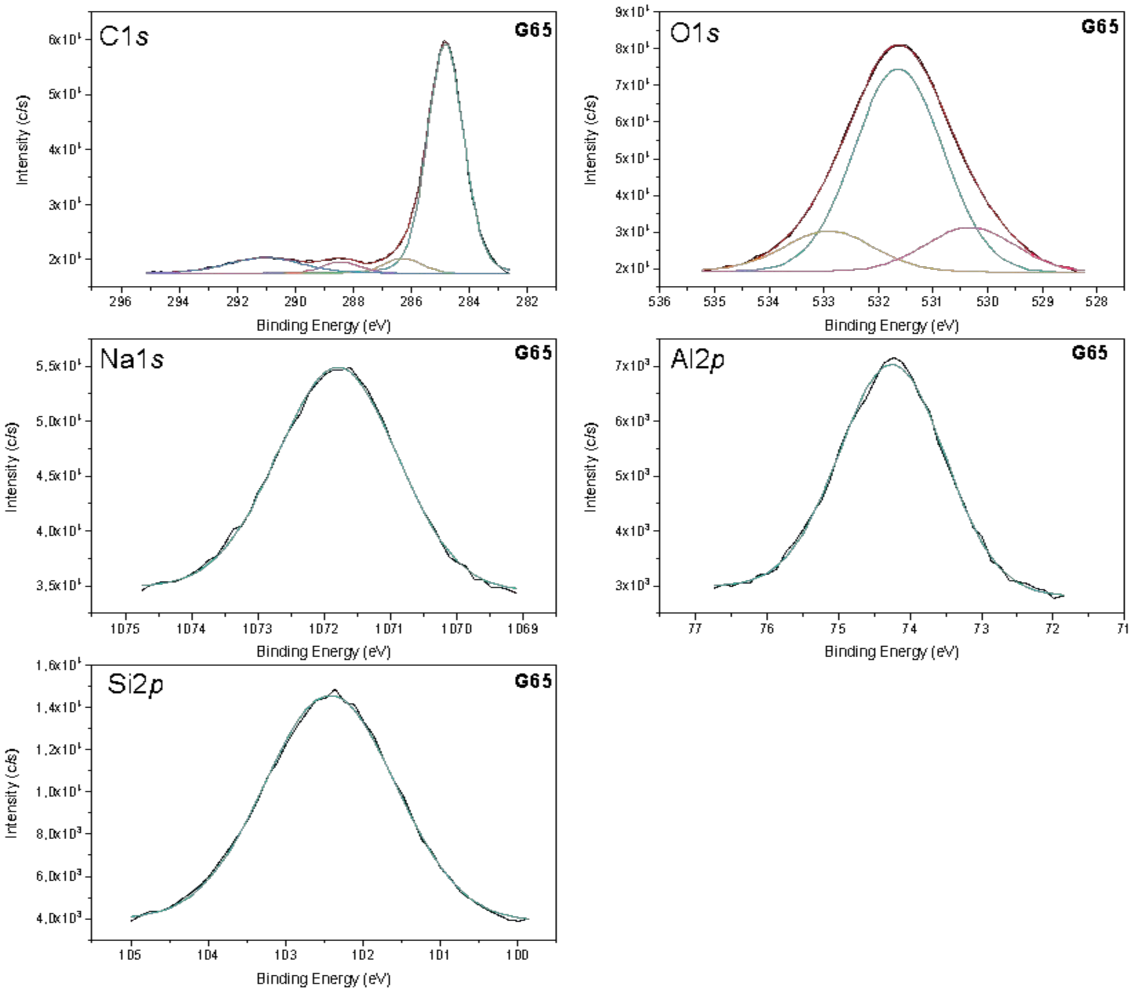

Figure S2. XPS decomposed atom electron orbitals spectra of the G65 sample.

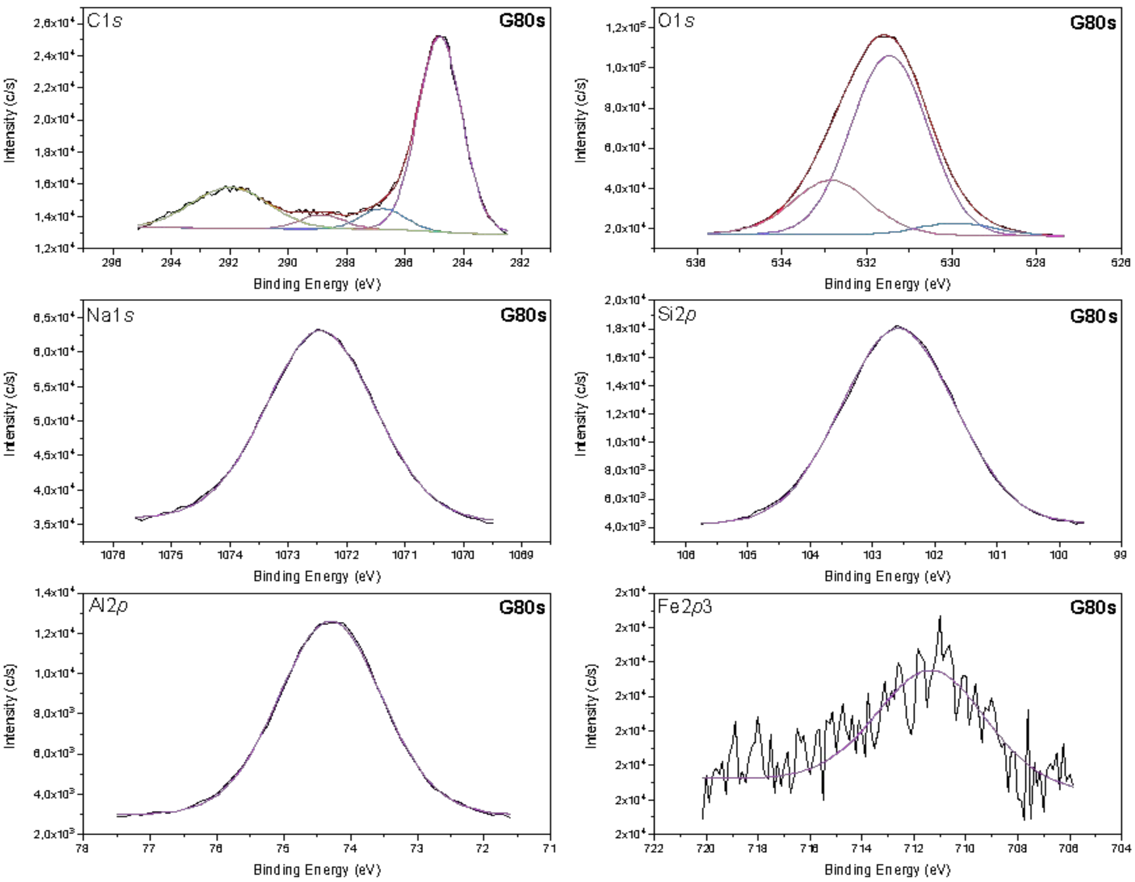

Figure S3. XPS decomposed atom electron orbitals spectra of the G80s sample.

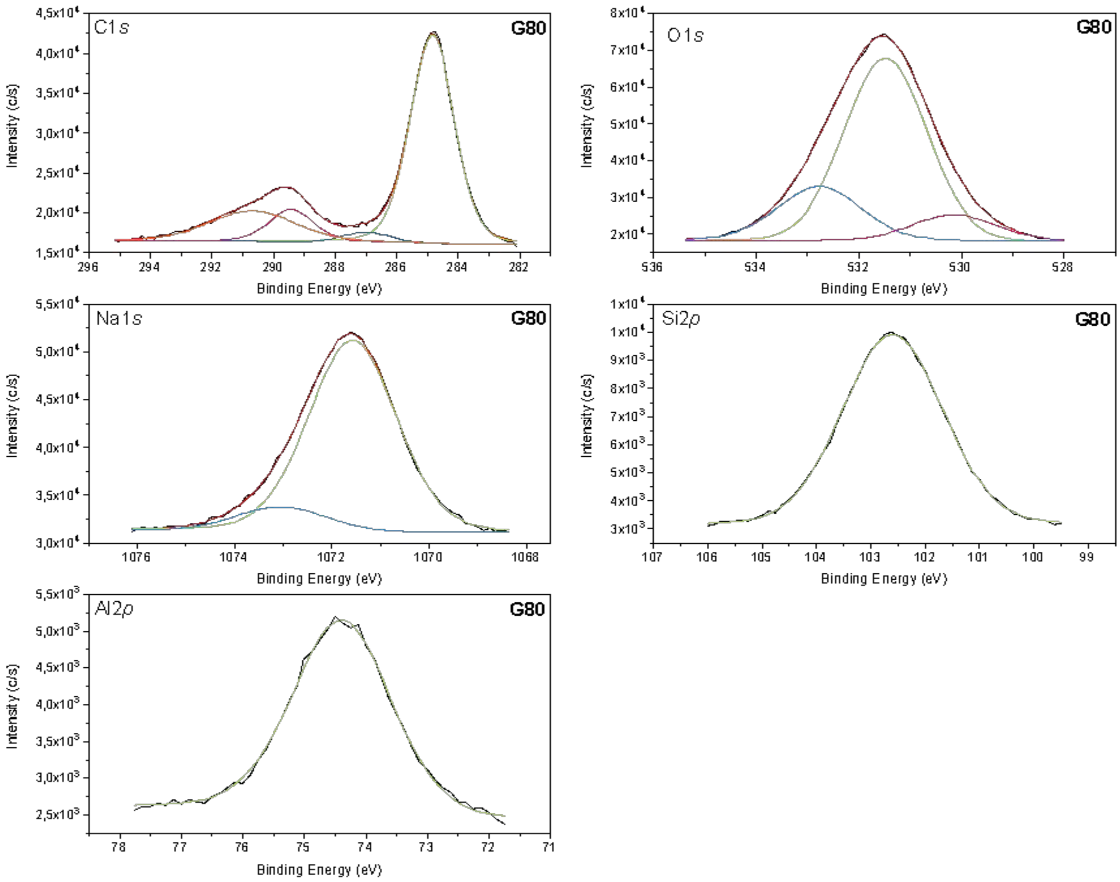

Figure S4. XPS decomposed atom electron orbitals spectra of the G80 sample.

Table S1: XPS quantification, area of the deconvoluted peaks of the samples.

	Area peaks (%)															
	G65s				G65				G80s				G80			
C1*s*	284.8 eV	286.4 eV	289.0 eV	291.3 eV	284.8 eV	286.3 eV	288.4 eV	291.0 eV	284.8 eV	286.7 eV	288.8 eV	292.0 eV	284.8 eV	287.0 eV	289.4 eV	290.7eV
	77.59	7.57	3.41	11.44	79.33	5.40	4.11	11.16	64.69	6.83	4.87	23.60	67.32	3.03	10.73	18.91
O1*s*	530.5 eV	531.5 eV	532.8 eV	-	530.3 eV	531.6 eV	532.9 eV	-	529.9 eV	531.5 eV	532.8 eV	-	530.1 eV	531.5 eV	532.8 eV	-
	9.63	67.05	23.32		15.41	70.15	14.44		5.12	71.97	22.90		9.67	69.08	21.25	
Na1*s*	1072.4 eV	-	-	-	1071.8 eV	-	-	-	1072.4 eV	-	-	-	1071.6 eV	-	-	-
	100				100				100				100			
Si2*p*	102.6 eV	-	-	-	102.4 eV	-	-	-	102.6 eV	-	-	-	102.6 eV	-	-	-
	100				100				100				100			
Al2*p*	74.3 eV	-	-	-	74.2 eV	-	-	-	74.3 eV	-	-	-	74.4 eV	-	-	-
	100				100				100				100			
Fe2*p*3	711.1 eV	-	-	-	-	-	-	-	711.0 eV	-	-	-	-	-	-	-
	100								100							

Table S2. Langmuir, Freundlich, Redlich Peterson, and Sips model parameters.

Gas	Temp.	Sample	Sips Model				Redlich-Peterson				Freundlich			Langmuir		
			K_S (mmHg)	q (mmol/g)	n_S	R^2	K_{RD} (mmHg)	q (mmol/g)	n_{RD}	R^2	K_F (mmHg)	n_F	R^2	K_L (mmHg)	q_{max} (mmol/g)	R^2
CO_2	30 °C	G65s	0.043	2.517	0.676	0.999	0.060	0.071	0.862	0.996	0.378	3.896	0.971	0.015	0.031	0.985
		G65	0.131	2.307	0.379	0.999	0.286	0.605	0.831	0.996	0.389	5.030	0.988	0.031	0.043	0.897
		G80s	0.048	2.758	0.682	0.999	0.068	0.063	0.881	0.998	0.455	4.049	0.965	0.017	0.039	0.984
		G80	0.065	1.159	0.590	0.999	0.047	0.148	0.839	0.995	0.162	3.790	0.973	0.018	0.017	0.979
	50 °C	G65s	0.015	2.325	0.761	0.999	0.020	0.032	0.834	0.999	0.138	2.634	0.981	0.007	0.013	0.995
		G65	0.034	1.855	0.515	0.999	0.045	0.282	0.727	0.998	0.106	3.010	0.993	0.008	0.009	0.971
		G80s	0.017	2.525	0.767	0.999	0.024	0.032	0.848	0.999	0.174	2.749	0.978	0.007	0.016	0.995
		G80	0.011	0.890	0.792	0.999	0.006	0.024	0.831	0.999	0.037	2.345	0.988	0.005	0.004	0.997
	100 °C	G65s	0.003	4.872	0.652	0.999	0.021	0.597	0.482	0.999	0.023	1.764	0.999	0.018	0.012	0.701
		G65	2.167E-4	6.394	0.269	0.990	2.238	1.633	0.761	0.968	0.139	4.188	0.968	0.002	0.003	0.993
		G80s	0.004	3.125	0.734	0.999	0.008	0.081	0.607	0.999	0.026	1.763	0.998	0.002	0.004	0.996

		G80	6.596E-5	3.220	0.464	0.997	3.530	7.560	0.654	0.995	0.047	2.887	0.995	0.007	0.004	0.916
CO	30 °C	G65s	3.227E-4	1.226	0.982	0.999	3.725E-4	0.001	0.840	0.999	6.53E-4	1.138	0.999	3.365E-4	1.076	0.999
		G65	7.473E-5	1.048	0.970	0.999	7.311E-5	0.011	0.434	0.999	8.856E-4	1.058	0.999	1.320E-4	0.511	0.999
		G80s	3.540E-4	0.887	0.996	0.999	3.084E-4	4.026E-4	0.983	0.999	5.680E-4	1.145	0.999	3.563E-4	0.863	0.999
		G80	2.580E-4	0.418	0.989	0.999	1.038E-4	6.497E-4	0.880	0.999	1.666E-4	1.112	0.999	2.689E-4	0.379	0.999
	50 °C	G65s	1.867E-4	1.200	0.992	0.999	2.179E-4	4.149E-4	0.898	0.999	3.136E-4	1.083	0.999	1.966E-4	1.097	0.999
		G65	4.144E-6	5.166	1.007	0.999	1.753E-4	5.042E-4	0.842	0.999	2.161E-5	0.995	0.999	1.466E-4	0.083	0.964
		G80s	1.552E-6	5.635	1.049	0.999	0.0126	1.453	0.049	0.999	8.628E-5	0.954	0.999	1.066E-4	1.099E-4	0.999
		G80	1.558E-4	0.314	0.991	0.999	4.739E-5	5.028E-4	0.851	0.999	6.459E-5	1.071	0.999	1.692E-4	0.276	0.999
	100 °C	G65s	9.811E-5	0.338	1.029	0.999	3.781E-5	5.941E-8	1.982	0.999	4.181E-5	1.019	0.999	5.174E-5	0.739	0.999
		G65	5.379E-6	0.018	2.022	0.987	2.210E-5	2.839E-8	1.262	0.977	2.401E-5	1.026	0.962	6.084E-9	0.008	0.964
H_2	30 °C	G65s	7.633E-6	2.894	0.983	0.997	0.004	1.575	0.020	0.997	2.241E-5	1.019	0.997	4.831E-9	0.007	0.955
		G65	4.357E-6	0.020	1.784	0.997	1.187E-5	2.361	1.424	0.996	7.140E-7	0.713	0.994	2.799E-9	0.003	0.952
		G80s	1.457E-4	0.091	0.984	0.997	1.848E-4	9.837	0.073	0.996	1.715E-5	1.074	0.996	1.710E-4	0.07	0.996
		G80	8.071E-7	6.402	1.083	0.996	0.017	3.297	0.083	0.995	5.187E-6	0.923	0.995	2.284E-9	0.003	0.953
